# Commentary: Serum Biomarkers Are Potential Diagnosis and Treatment Targets for Depressive Symptoms in Patients With Cardiovascular Diseases

**DOI:** 10.3389/fpsyt.2021.649705

**Published:** 2021-06-07

**Authors:** Cunming Liu, Chun Yang

**Affiliations:** Department of Anesthesiology and Perioperative Medicine, The First Affiliated Hospital of Nanjing Medical University, Nanjing, China

**Keywords:** Cardiovascular diseases, depression, biomarkers, treatment, diagnosis

Cardiovascular diseases (CVDs) and depression are the major causes for death and disability worldwide (1, 2). Currently, depression has been confirmed as an independent risk factor for CVDs (3). However, the comorbidity of CVDs and depression still lacks effective objective diagnostic indicators. This is the question that Wu et al. (4) tried to answer in this issue of *Frontiers in Psychiatry* (*Interplay of Stress, Pain and Psychiatric Diseases*). The authors recruited 116 patients with stable coronary artery disease (SCAD) and 45 healthy controls. According to the Zung Self-Rating Depression Scale (SDS), the SCAD patients were divided into a depressive group and a non-depressive group, and measured serum levels of fibroblast growth factor 21 (FGF21), β-klotho, mature brain-derived neurotrophic factor (mBDNF), and BDNF precursor (BDNF precursor, proBDNF). The findings showed that depression score was positively correlated with the severity of SCAD, and that serum FGF21, β-klotho, mBDNF, and proBDNF levels were linked to depressive symptoms in SCAD patients.

As an available approach to diagnose depression, clinical face-to-face interviews still have the following problems. Firstly, to facilitate comparison, physicians try to promote specific tools to evaluate depression in patients with CVDs. Still, many different questionnaires are adopted in a large number of various studies to screen patients. Differential standards for selecting evaluation scales may result in an evitable bias in results and conclusions. Secondly, depressive symptoms in patients with CVDs are often firstly recognized by the cardiologists. Unfortunately, some cardiologists have not yet been professionally trained in psychiatry. The non-professional background may affect the efficacy of scale evaluation. Thirdly, a few patients with CVDs are severely ill at hospital admissions, for example with acute myocardial infarction, acute heart failure, and even multiple organ failure. It seems difficult to develop a questionnaire for these patients. Therefore, it is necessary to find objective detection indicators, such as serum biomarkers, to assess the relationship between CVDs and depression comorbidities, or the severity of depressive symptoms in CVDs patients.

FGF21 is a member of the FGF family and is expressed in multiple organs, including liver, kidney, fat cells, and cardiomyocytes. FGF21 can specifically bind to the co-receptor β-klotho and release into the circulation (5), and the levels of FGF21 are significantly affected by β-klotho (6). BDNF is an essential member of the neurotrophic factor family, regulating the development and plasticity of neurons. Notably, BDNF as a clue to explore potential biomarkers of cardiovascular diseases comorbid depression has been reported (4, 7). Plasminogen activator inhibitor-1 (PAI-1) could act as a mediator of depression-induced CVDs through sleep disorder, adiposity, BDNF metabolism, systemic inflammation, and hypothalamic-pituitary-adrenal (HPA) axis dysregulation. Similarly, sigma-1 receptor chaperone plays a vital role in CVDs comorbid with depression (8). Sigma-1 receptor agonists such as endogenous neurosteroid dehydroepiandosterone (DHEA) and selective serotonin reuptake inhibitors (SSRIs) have shown potent cardio-protective and antidepressant effects. In this study, Wu et al. (4) firstly found that serum β-klotho in the depression group was significantly reduced, and that β-klotho was negatively correlated with the depression score. However, the detailed mechanisms remain to be identified. Also, Wu et al. (4) found that serum mBDNF levels were decreased in SCAD patients, and that mBDNF level and mBDNF/proBDNF ratio in the depression group were significantly decreased. More importantly, serum mBDNF levels were negatively correlated with SDS score. Subgroup analysis found that the incidence of depression in the low-level mBDNF group was higher than that of the high-level mBDNF group, suggesting that increasing serum mBDNF levels may improve the depression symptoms of SCAD patients. However, there are several limitations in the study. The study did not discuss confounding factors that might affect the results of the study, such as education, income, social support, and marital status. In addition, this is a phenomenological study that only observed the differences of these biomarkers in the SCAD and SCAD with depressive symptoms groups. The authors did not delve into the possible mechanisms underlying the differences in biomarkers. Furthermore, the authors did not discuss the sensitivity and specificity of these biomarkers for the diagnosis of depressive symptoms in patients with CVDs.

Sir William Harvey, 350 years ago, first observed that negative emotions have a deleterious effect on the heart (9). Since the late 1980s, research directions have gradually shifted from psychiatric patients with depression to the high-risk factors for depression in patients diagnosed with CVDs in the community, aiming to explore the impact of depression on CVDs (10). Unfortunately, most studies still maintain “depression” and “CVDs” as two separate diseases, lacking an accurate definition for “psycho-cardiology.” Therefore, it is difficult for doctors to distinguish the sequence of the occurrence and development of depression and CVDs, and to study the causal relationship between depression and CVDs. Another difficulty is that the mechanisms underlying the co-morbidity of CVDs and depression are complex and still not fully understood. In addition to traditional mechanisms, novel mechanisms such as gut microbiota, endocrine signaling, microRNAs have also been extensively studied (11). This might propose additional challenges to understand the pathogenesis and therapeutic mechanisms of the comorbidity. In addition to potential confounding factors, including smoking, diabetes, obesity, and sedentary lifestyle, further long-term studies are needed to track the relationship between CVDs, mood, depression, and psychosocial status. Considering the different focus of diagnosis and treatment by different specialists, diagnosing and treating such patients in a multidisciplinary manner is necessary. Moreover, reliable testing indicators, such as serum biomarkers, need to be established to evaluate depressive symptoms and CVDs and the diagnosis, treatment, and prognosis of the comorbid patients. Besides, it seems inappropriate for several studies to simply classify patients as depressed or non-depressed through the depression scale. Evidence has shown a correlation between the severity of depression and cardiac risk, and even the appearance of mild symptoms will bring risks (12). However, patients with mild depressive symptoms may be classified as non-depressed based on the widely used depression questionnaires. Consequently, artificial categorization of depression severity scores (for example, non-depressed vs. depressed) may weaken the strength of the correlation between depression severity and cardiac prognosis.

In summary, depression as one of the independent cardiovascular risk factors has a significant impact on the public health. The paper by Wu et al. (4) has raised the necessary attention to seek biomarkers for CVDs and depression comorbidity. Future studies should better understand the bidirectional relationship between CVDs and depression and design more rigorous long-term follow-up research programs to find effective biomarkers in a larger sample.

## Author Contributions

All authors listed have made a substantial, direct and intellectual contribution to the work, and approved it for publication.

## Conflict of Interest

The authors declare that the research was conducted in the absence of any commercial or financial relationships that could be construed as a potential conflict of interest.
